# A Case of Angioimmunoblastic T-cell Lymphoma That Mimics As Autoimmune Diseases and Infections

**DOI:** 10.7759/cureus.16439

**Published:** 2021-07-17

**Authors:** Kaku Kuroda, Ajay Tambe, Rahila Iftikhar, Amitpal S Nat, Alina Basnet

**Affiliations:** 1 Family Medicine, State University of New York (SUNY) Upstate Medical University, Syracuse, USA; 2 Hematology and Oncology, State University of New York (SUNY) Upstate Medical University, Syracuse, USA; 3 Internal Medicine, State University of New York (SUNY) Upstate Medical University, Syracuse, USA

**Keywords:** small vessel vasculitis, autoimmune like, ecchymosis, maculopapular rash, angioimmunoblastic t-cell lymphoma

## Abstract

Angioimmunoblastic T-cell lymphoma (AITL) is an aggressive malignancy with a presentation like either autoimmune diseases, drug reactions, or infections. We hereby present a unique case of AITL. A 61-year-old Caucasian male with a past medical history of chronic obstructive pulmonary disease (COPD) presented to the emergency department with a rash over his bilateral knees, shortness of breath, and productive cough of few days. He was managed for suspected COPD exacerbation associated with community-acquired pneumonia. On the day of admission patient was having an itchy maculopapular rash, ecchymosis on the left flank, and generalized lymphadenopathy. Physical exam showed generalized lymphadenopathy. Laboratory tests revealed leukocytosis, thrombocytopenia and were positive for multiple autoantibodies. Epstein-Barr virus polymerase chain reaction and hepatitis B virus core antibody were positive. Skin biopsy revealed findings suggestive of a small vessel vasculitis. Inguinal lymph node biopsy showed AITL. The patient recovered with chemotherapy.

The case illustrates that clinical presentation of AITL mimics rheumatologic disorders and infections. This complexity could arise from the follicular T helper cell, which is an important checkpoint for B cell activation and differentiation. Additionally, skin involvement is one of the important findings of AITL and a variety of lesions have been reported as skin manifestations.

## Introduction

Angioimmunoblastic T-cell lymphoma (AITL) is a subtype of mature peripheral T-cell lymphoma. It is characterized as polymorphic lymph nodal lymphoid infiltrate along with the prominent proliferation of endothelial venules and follicular dendritic cells [1]. AITL is an aggressive malignancy with a presentation like either autoimmune diseases, drug reactions, or infections. We came across a unique case of AITL who presented primarily with skin rash, positive autoimmune antibodies and, mimicking rheumatological disorder.

## Case presentation

A 61-year-old Caucasian male presented to the emergency department with a rash over his bilateral knees, shortness of breath, and productive cough of few days. His past medical history was significant for chronic obstructive pulmonary disease (COPD), hypertension, hypercholesterolemia, and benign prostatic hyperplasia. Surgical history was significant for mitral valve repair for mitral stenosis due to rheumatic heart disease. His medications included ramipril, atorvastatin, and tamsulosin. He had 40-pack years of smoking and denied alcohol intake and illicit drug use. Family history included stroke and heart disease in his father.

**Figure 1:**
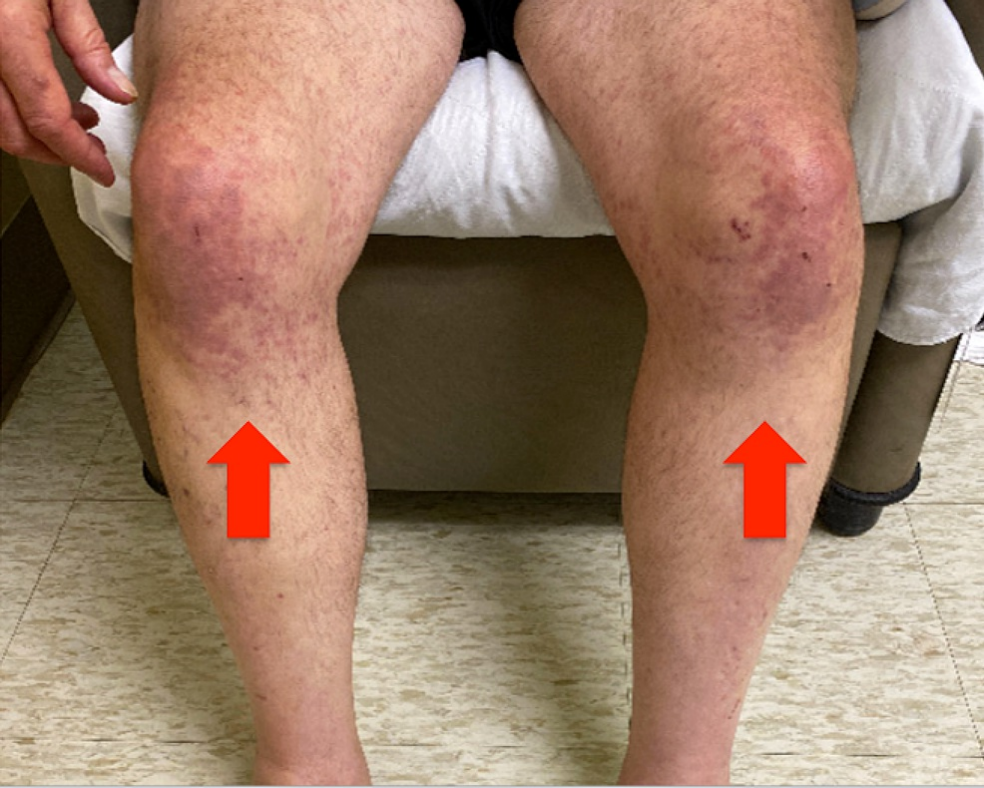
Erythematous maculopapular rash over bilateral knees (red arrow)

Physical examination revealed cervical lymphadenopathy, bilateral wheezing with accessory muscle use, and an erythematous maculopapular rash over bilateral knees (Figure 1).

Initial laboratory data showed leukocytosis and mild hyponatremia. Chest x-ray revealed patchy opacities on the left lung base. An echocardiogram showed moderate mitral annular calcification and mild aortic valve sclerosis with trivial aortic regurgitation, otherwise, no significant findings were noted. Doppler ultrasound for the lower extremity showed no evidence of deep vein thrombosis. The patient was managed for suspected COPD exacerbation associated with community-acquired pneumonia. He was started on inhaled short-acting beta-adrenergic agonists, short-acting muscarinic antagonists, an intravenous corticosteroid, intravenous ceftriaxone, and azithromycin. On the next day of admission, white blood cell count went up to 15,200, and platelet level was decreased to 99,000 (Table 1).

**Table 1:** Laboratory findings on the second day of admission Abbreviations: WBC: white blood cell; Neutro: neutrophil; Lym: lymphocyte; Hb: hemoglobin; Hct: hematocrit; MCV: mean cell volume; Plt: Platelet; BUN: blood urea nitrogen; Crn: creatinine; Pro BNP: pro B-type natriuretic peptide.

Variable	Value		Variable	Value	
WBC	15,200	10^3^/mcL	BUN	20	IU/L
Neu	82.0	%	Crn	0.87	IU/L
Lym	7.0	%	Na	132	mg/dL
Hb	14.8	g/dL	K	4.3	mg/dL
Hct	43.7	%	Cl	98	mg/dL
MCV	91.9	%	Ca	8.7	mg/dL
Plt	99	10^3^/mcL	Pro BNP	295	pg/mL

Despite the treatment, the patient had persistent symptoms and leukocytosis. Chest computed tomography angiography (CTA) was obtained and revealed multiple pulmonary nodules and enlarged mediastinal lymph nodes. His erythematous maculopapular rash on bilateral knees started to spread over his back, bilateral upper extremities, and feet with pruritus. Initially, drug reaction was suspected, and all antibiotics were discontinued. However, the rash did not resolve, and a skin biopsy was performed. Skin pathology revealed perivascular lymphocytic inflammation and thickened vascular endothelial cells, suggestive of a small vessel vasculitis. Additional laboratory tests showed positive antinuclear antibodies with 1:160 for speckled patterns and hypocomplementemia. Autoimmunologic workup was found positive for anti-SSA/Ro antibody, anti-SSB/La antibody, anti-Jo-1 antibody, anti-RNP antibody, anti-Histone antibody, and rheumatoid factor (Table 2). 

**Table 2:** Additional laboratory findings *Abbreviations: ANA; antinuclear antibodies; Anti-dsDNA: anti-double strand DNA antibodies; Anti-Sm: anti-Smith antibodies; Anti-SSA/Ro: anti-Sjögren's syndrome related antigen A autoantibodies; Anti-SSb/La: anti-Sjögren’s syndrome related antigen B antibodies; Anti Scl-70: anti-Scl-70 autoantibodies; Anti-Jo-1: anti-Jo-1 antibodies; Anti-RNP: anti-ribonucleoprotein antibodies; Anti-Centromere: anti-Centromere antibodies; Anti-Histone: anti-Histone antibodies;  RF: rheumatoid factor; Anti-CCP: anti-cyclic citrullinated peptide; PR3-ANCA: proteinase 3 antineutrophil cytoplasmic antibodies; MPO-ANCA: myeloperoxidase antineutrophil cytoplasmic antibodies; TSH: thyroid stimulating hormone; PSA: prostate specific antigen; ACE: angiotensin converting enzyme; LDH: lactic acid dehydrogenase; CRP: C reactive protein; ESR: erythrocyte sedimentation rate; HAV IgM: hepatitis A virus IgM; HBsAg; hepatitis B surface antigen; HBeAg; hepatitis B virus e antigen; HB Core IgM: hepatitis B core IgM; HBV DNA: hepatitis B virus DNA; HCV IgM: hepatitis C virus IgM; HIV: human immunodeficiency virus;  EBV VCA IgM: Epstein-Barr virus viral capsid antigen IgM; EBV DNA: Epstein-Barr virus DNA.

Variable	Value		Variable	Value	
ANA (Speckled)	160	1/dilution	C3	56	mg/dL
Anti-dsDNA	(-) 57	IU/mL	C4	4	mg/dL
Anti-Sm	(-) 41	IU/mL	TSH	0.868	mg/dL
Anti-SSA/Ro	(+) 120	IU/mL	PSA	1.3	ng/mL
Anti-SSb/La	(+) 264	IU/mL	ACE	62	U/L
Anti Scl-70	(-) 41	IU/mL	LDH	550	IU/L
Anti-Jo-1	(+) 190	IU/mL	CRP	2.5	mg/dL
Anti-RNP	(+)114	IU/mL	ESR	31	mm/h
Anti-Centromere	(-) 74	IU/mL	Syphilis IgM/IgG	Negative	
Anti-Histone	(+) 174	IU/mL	HAV IgM	Non reactive	
RF	(+) 56	IU/mL	HBsAg	Negative	
Anti-CCP	(-) <0.5	IU/mL	HBeAg	Negative	
Anti-cardiolipin IgM	(-) 9.4	IU/mL	HB Core IgM	Reactive	
Anti-cardiolipin IgG	(-) 4.4	IU/mL	HBV DNA	Not detected	
Lupus Anticoagulant	(-)		HCV IgM	Non-reactive	
Cryoglobulin	(+)		HIV	Negative	mg/dL
PR3-ANCA	(+) 20.8	CU	EBV VCA IgM	(+) 4.81	
MPO-ANCA	(-) <5.0	CU	EBV DNA	49,999	copies/mL

Hepatitis B virus (HBV) core antibody was positive however HBsAg, HBeAg, HBV DNA, and anti-HBs were not detected. Epstein-Barr virus (EBV) viral capsid antigen IgM antibody was positive, and EBV polymerase chain reaction (PCR) was positive. Differential diagnoses included vasculitis, sarcoidosis, late-onset systemic lupus erythematosus, Sjögren's syndrome, dermatomyositis, mixed connective tissue disease, lung cancer with paraneoplastic syndrome, Steven-Johnson syndrome, and lymphoma.

**Figure 2:**
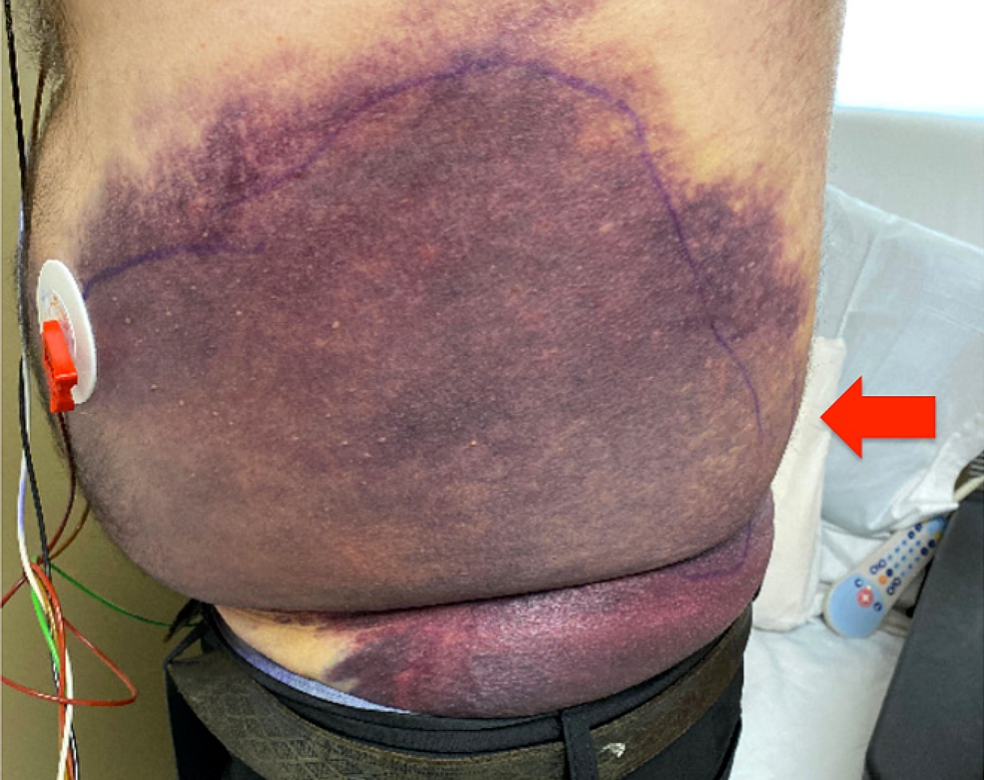
Ecchymosis on his left side of the abdominal wall (red arrow)

He went on to develop ecchymosis on his left flank with thrombocytopenia (Figure 2).

**Figure 3:**
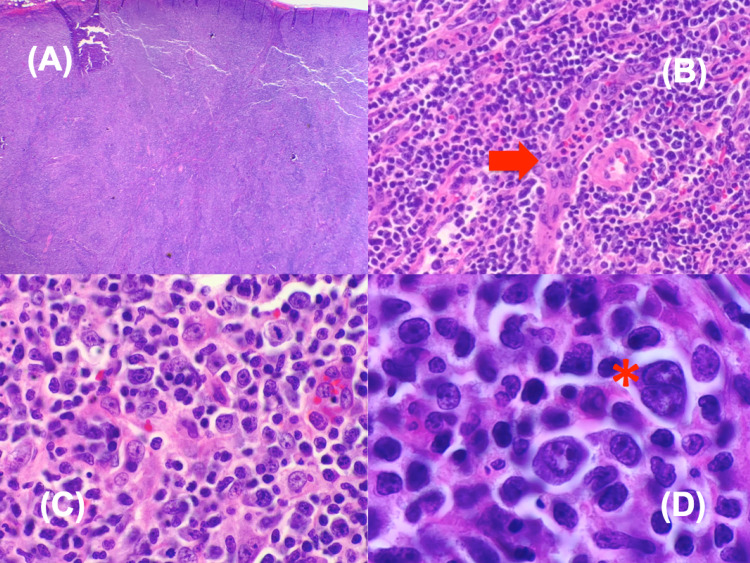
Inguinal lymph node biopsy (A) The lymph node architecture was largely effaced by a polymorphous lymphoid infiltrate. (B) Prominent vasculature with arborizing high endothelial venules is noted (red arrow). (C) Pleomorphic medium-sized cells with irregular nuclei, abundant pink cytoplasm, irregular nuclei, and prominent nucleoli. (D) Reed-Sternberg-like cells are also present (asterisks), with occasional binucleation.

Subsequently, low-molecular-weight heparin initially started on admission for thromboprophylaxis was discontinued. Workup revealed a negative heparin-induced thrombocytopenia panel and enlarged inguinal lymph nodes bilaterally. Inguinal lymph node biopsy showed AITL (Figures 3A-3D).

**Figure 4:**
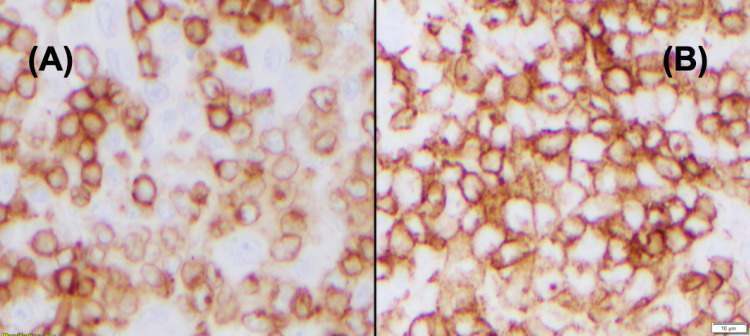
Immunohistochemical staining of the inguinal lymph node (A) CD3: staining is faint in the neoplastic cells and strong in the small round cells, consistent with reactive T-cells. (B) CD10: The same cells highlighted by CD3 are also positive for CD10.

Immunohistochemical staining of the inguinal lymph node biopsy demonstrated diffusely positive CD3 and CD10 (Figures 4A, 4B).

T-cell receptor gamma chain gene-PCR assay revealed a clonal T-cell receptor gamma chain gene rearrangement, consistent with an abnormal proliferative process. There was no evidence of a monoclonal population of cells exhibiting either an Immunoglobulin Heavy Chain or Kappa Light chain gene rearrangement. Therefore, the abnormal B-cell lymphoproliferative process was excluded. Bone marrow aspiration and biopsy showed minimal bone marrow involvement by AITL. Chromosome analysis showed a normal male karyotype.

The patient received chemotherapy with cyclophosphamide, doxorubicin, etoposide, and brentuximab vedotin (BV), with prednisone. Following this treatment, the patient developed spontaneous left-sided pneumothorax, subsequently leading to left pneumohemothorax with hypovolemic shock. The later complications required ICU stay, left thoracotomy, diaphragmatic repair, and partial rib resection. After the prolonged hospital stay, he recovered and was subsequently discharged to rehabilitation. Due to the prolonged hospital stay with the first cycle of multi-agent chemotherapy and significant clinical deterioration, a plan was made to treat him subsequently with only single-agent BV. He has received 11 cycles of BV with pegfilgrastim support. The CT thorax, abdomen, and pelvis after 11 cycles of BV continued to show ongoing response to the treatment. The patient himself whose initial Eastern Cooperative Oncology Group (ECOG) performance status on presentation was 3 has improved to 1 with therapy.

## Discussion

AITL is an aggressive malignancy that is the second most common T-cell lymphoma in Western countries. The three-year overall and progression-free survival rates were reported as 54% and 38%, respectively, in a retrospective study with AITL with a median follow-up of 42 months [2]. The symptoms of AITL are not specific and, consequently, the diagnosis can be challenging and sometimes delayed. The clinical features of AITL are more related to immune dysfunction than to tumor growth itself. Immunohistochemical staining of the lymph nodes and cutaneous specimens can help differentiate AITL from other peripheral T-cell lymphomas.

Patients commonly present with the acute onset of a systemic illness characterized by generalized lymphadenopathy, B-symptoms (fevers, unintentional weight loss, and/or drenching night sweats), and hepatosplenomegaly [1,3]. Skin involvement is one of the important findings and is seen in up to 50% of patients [4,5]. A variety of skin lesions has been reported such as purpura, erosion, urticaria, reticular rash, nodules [3,6-8]. The common laboratory findings include elevated lactate dehydrogenase level and erythrocyte sedimentation rates, lymphopenia, anemia, and thrombocytopenia [1-3]. Immunologic abnormalities like polyclonal hypergammaglobulinemia and positive Coombs test can be associated [1-3]. A retrospective study of AITL revealed the presence of antinuclear antibodies in 28% of patients [2]. Additionally, EBV has been reported to be associated with AITL [1].

Our patient’s presentation mimics autoimmune diseases such as late-onset systemic lupus erythematosus, Sjögren's syndrome, dermatomyositis, and mixed connective tissue disease. The presence of thrombocytopenia, malar rash, generalized lymphadenopathy, and hypocomplementemia prompted us to look for autoimmune diseases. Multiple autoimmune antibodies were found to be positive in this case including ANA, anti-SSA/SSB antibodies, anti-Jo-1 antibody, anti-RNP antibody. It is known that ANA can be present in some cases with AITL and AITL can mimic systemic lupus erythematosus [9], but there are no data about the positivity of other autoantibodies including anti-SSA/SSB antibodies, anti-Jo-1 antibody, anti-RNP antibody in AITL as seen in our case. This positivity for multiple autoantibodies could arise from the follicular T helper cell as the cell of origin, which is an important checkpoint for B-cell activation and differentiation [10]. Our patient tests positive for anti-viral capsid antigen IgM for EBV and hepatitis B core antibody. While EBV has been reported to be associated with AITL [1], an association between AITL and HBV has not been described in previous studies. Although immunodeficiency and opportunistic infections are thought to stem from immune dysregulation of both normal T-cells and B-cells, HBV DNA was not detected in our case, which indicates no active infection. These positive antibodies were thought to be immunological cross-reaction due to the proliferation of abnormal follicular T-cells.

Finally, our patient developed an erythematous maculopapular rash that started on bilateral knees and went on to spread over his back, bilateral upper extremities, and bilateral feet with pruritus. In previous studies, it is reported that rash is often pruritic and in the form of a nonspecific generalized maculopapular rash, which resembles a viral exanthema or a drug reaction [3,6]. A skin biopsy should be considered in such a complicated case. In the present case, the findings of skin biopsy are suggestive of small vessel vasculitis. In a previous retrospective study, vasculitis was found in 12% of patients with AITL in 77 cases [11]. Circulating immune complexes or a cell-mediated immune response have been implicated as possible pathogenesis of paraneoplastic small vessel vasculitis in lymphoproliferative disorders [12]. In addition to the malar rash, big left abdominal wall ecchymosis was seen in this case. It is assumed that the cause of this ecchymosis is multifactorial, including vasculitis and thrombocytopenia.

## Conclusions

We experienced a unique case of AITL mimicking rheumatological disorder and infections. This complexity could arise from the follicular T helper cell, which is an important checkpoint for B-cell activation and differentiation. Additionally, the skin manifestation of AITL may include a variety of lesions.
